# Response Characteristics Study of Ethylene Sensor for Fruit Ripening under Temperature Control

**DOI:** 10.3390/s23115203

**Published:** 2023-05-30

**Authors:** Xiaoshuan Zhang, Yuliang Li, Tianyu Hong, Srdjan Tegeltija, Mladen Babić, Xiang Wang, Gordana Ostojić, Stevan Stankovski, Dragan Marinković

**Affiliations:** 1 College of Engineering, China Agricultural University, Beijing 100083, China; zhxshuan@cau.edu.cn (X.Z.); lyl@cau.edu.cn (Y.L.); 2019307070205@cau.edu.cn (T.H.); wxzrjj@cau.edu.cn (X.W.); 2 Center for Identification Technology, Faculty of Technical Sciences, University of Novi Sad, Trg Dositeja Obradovica 6, 21000 Novi Sad, Serbia; mladen.babic@uns.ac.rs (M.B.); goca@uns.ac.rs (G.O.); stevan@uns.ac.rs (S.S.); 3 Faculty of Mechanical Engineering, University of Niš, Aleksandra Medvedeva 14, 18000 Niš, Serbia; dragan.marinkovic@tu-berlin.de; 4 Faculty of Mechanical Engineering and Transport Systems, TU Berlin, Str. d. 17. Juni 135, 10623 Berlin, Germany

**Keywords:** fruit, ethylene sensor, response characteristics, cold-chain logistics

## Abstract

Post-ripening fruits need to be ripened to reach edible conditions, as they are not yet mature enough when picked. Ripening technology is based mainly on temperature control and gas regulation, with the proportion of ethylene being one of the key gas regulation parameters. A sensor’s time domain response characteristic curve was obtained through the ethylene monitoring system. The first experiment showed that the sensor has good response speed (maximum of first derivative: 2.01714; minimum of first derivative: −2.01714), stability (xg: 2.42%; trec: 2.05%; Dres: 3.28%), and repeatability (xg: 20.6; trec: 52.4; Dres: 2.31). The second experiment showed that optimal ripening parameters include color, hardness (Change Ⅰ: 88.53%, Change Ⅱ: 75.28%), adhesiveness (Change Ⅰ: 95.29%, Change Ⅱ: 74.72%), and chewiness (Change Ⅰ: 95.18%, Change Ⅱ: 74.25%), verifying the response characteristics of the sensor. This paper proves that the sensor was able to accurately monitor changes in concentration which reflect changes in fruit ripeness, and that the optimal parameters were the ethylene response parameter (Change Ⅰ: 27.78%, Change Ⅱ: 32.53%) and the first derivative parameter (Change Ⅰ: 202.38%, Change Ⅱ: −293.28%). Developing a gas-sensing technology suitable for fruit ripening is of great significance.

## 1. Introduction

With the rapid development of the economy and the fruit industry, the consumption of imported fruits in China is increasing, and people are paying more attention to the nutritional value of fruits [1,2]. Post-ripe fruits account for a large part of this growth, and grow mainly in southern China due to its climatic and geographical conditions. Whether the fruits are transported from the south to the north or imported from abroad, it takes a long time to reach the market, so ensuring their freshness is a major issue. The postharvest logistics process of post-ripe fruits is divided into several steps. These include picking, pre-cooling, transportation, storage, ripening, transportation, supermarkets, and other links in which cold-chain logistics plays a very important role [3,4,5,6].

Post-ripe fruits with high nutritional value have been studied, including banana, avocado, kiwi, mango, and many others [7,8]. The storage period of post-ripe fruits is very short when they are fully ripe, so they are typically picked at a low stage of ripeness. However, the fruit is hard, low in soluble solids, and of poor edible condition. Therefore, immature fruit must ripen naturally or artificially to reach an edible state. The fruit’s natural ripening has problems, such as long ripening time and poor quality of taste, which are inconvenient for consumers. Therefore, postharvest ripening is very important for peeled and post-ripe fruits. Ripening technology is mainly based on temperature control and gas conditioning, an indispensable part of the ripening fruit supply chain.

The mainstream ripening techniques nowadays include ripening directly with ethylene, controlled ripening with corresponding reagents (ethephon, 1-MCP), and controlled ripening by building mathematical models to characterize ripening characteristics. For the first of these, ethylene is used to accelerate the metabolism process and cause a large change in the characteristics of the internal components. Ripening can be effectively accelerated with 100 ppm ethylene at 20–23 °C by controlling ethylene, CO_2_, and O_2_ conditions [9,10,11,12]. When using corresponding reagents to control ripening, ethephon, ethylene-mbs, and 1-MCP significantly affect the regulation of biological substances, though their effects on different fruits vary and, while they are commercially available, they are more complex and thus limited [13,14,15]. Finally, with regard to the building of mathematical models to characterize ripening characteristics, Nadya et al. were able to predict the ripening characteristics of cheese by modeling with partial least squares and artificial neural networks [16]. A survey has found that fruit enterprises mostly set ripening storage at 17–20 °C, put the ethylene generator in the storage room, set its working time to about 10 h, and then exhaust the ethylene gas. After that, the temperature and humidity adjustments to ripen the fruit are undertaken manually. In summary, the current ripening industry lacks an accurate measure of ethylene to control the ripening of fruits. Therefore, it is necessary to accurately monitor ethylene during ripening for post-ripe fruits.

The factors affecting the ripening effect are ethylene, temperature, CO_2_, etc. The most important factors are ethylene and temperature. Temperature is easy to detect and control under the existing technology. However, due to its limited chemical function, the detection of ethylene at low concentrations has become an important problem [17]. The literature notes ethylene detection methods such as gas chromatography and electrochemical, optical, and chemical methods, but these are mostly characterized by a large range and low accuracy, which cannot meet the demand for a small range and a high accuracy in the ripening processes [18,19,20].

To address the above issues, based on the selection of an ethylene detection sensor suitable for fruit ripening, this paper presents a comprehensive analysis via the extraction of the static and dynamic characteristics of a gas sensor that is widely used in practical applications. The steady-state response curve of the ethylene sensor to the target gas was tested to extract the parameters determining the relevant steady-state and dynamic characteristics, which were also analyzed and discussed [21]. Finally, these analyses were verified by an actual fruit ripening test (avocado as a trial use case) that proves that the sensor has good performance and can meet the needs of ethylene gas monitoring in fruit ripening.

## 2. Experimental Protocol and Method

### 2.1. Feature Parameters and Extraction Method

**Figure 1:**
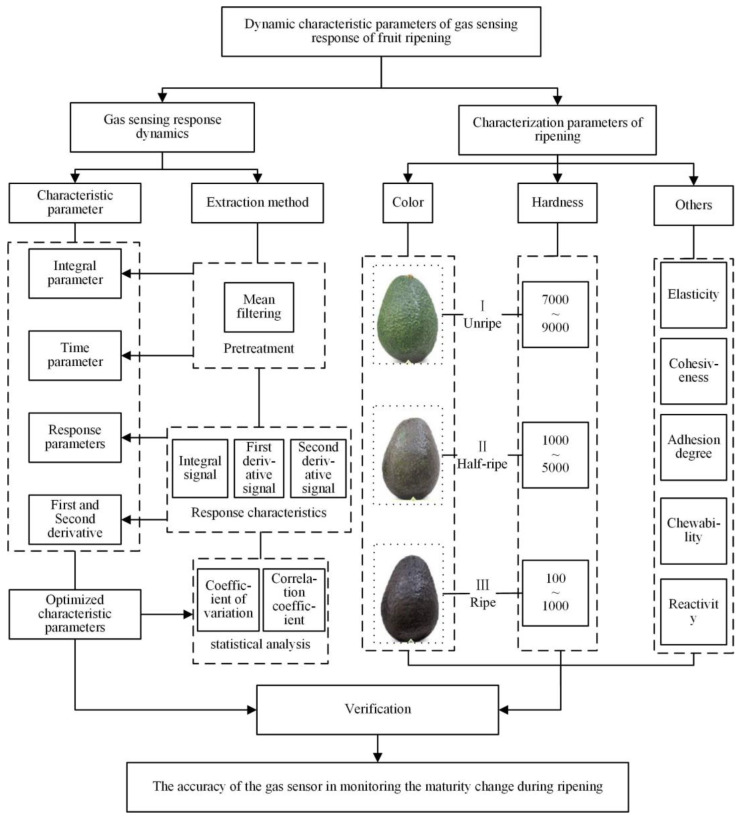
Test method flow chart.

This paper focuses on the dynamic characteristic parameters of gas sensor response and indicates the ripening effects of the ripening characterization parameters. The experimental method is shown in Figure 1. The kinetic characteristic parameters were obtained by different extraction methods (mean filter, differential) and the ripening state of the avocado was characterized by its color and hardness at different ripening stages. Some other indicators (hardness, elasticity, cohesiveness, adhesion, chewability, and reactivity) also changed. Finally, the accuracy of the gas sensor in monitoring the ripening process was verified by analyzing the correlation between the kinetic parameters of the gas sensing response and the ripening characterization parameters.

The characteristics of a sensor are usually described by the characteristic parameter method. The typical parameters shown in Figure 1 are important indicators by which to characterize the gas sensor and include response, derivative, time, etc. Table 1 gives a detailed description of some typical parameters [22,23,24,25,26]. After obtaining the time domain sensing signals, data preprocessing is required to eliminate gross errors and outliers caused by environmental factors (e.g., vibration, temperature, humidity, and other gases) during the detection process. This paper adopts the mean filtering method for data preprocessing because the mean filtering method can effectively eliminate periodic disturbances with high accuracy, good stability, and easy implementation [27,28,29].

Relevant response characteristics such as steady-state response value, maximum response value, final recovery value, response time, and recovery time can be extracted directly from the response curve of the gas sensor [30]. Furthermore, from the derivative and integration curves, the characteristics of the gas sensor response, such as the rate of change and the amplitude accumulation can be extracted [31,32,33]. Because all data are measured under the same conditions, the extracted characteristic parameters could be considered to comprise a Gaussian distribution. Their degree of variation is compared by calculating each parameter’s mean, variance, standard deviation, and coefficient of variation. Additionally, the parameters with small variations were selected to characterize the performance of the sensor and communication data transfer [34,35,36,37,38,39,40,41].

A large correlation could exist between the characteristic parameters that are listed in Table 1. Therefore, the correlation between the parameters needs to be analyzed to extract the best parameters to characterize the response characteristics of the ethylene sensor. In this paper, Pearson’s correlation coefficient was used to describe the closeness of the connection between each covariate.

Figure 1 demonstrates the ripening characterization process which evaluates the maturity level of a specific fruit, in this case, the avocado, to estimate its stage of ripening. The parameters used to characterize the ripening of the avocado, as referenced in [42], include aspects such as color and hardness, among other factors. Here, the avocado is used as an example, and other fruits that require postharvest ripening have similar ripening characterization parameters. The study classified avocados into three classes according to their ripeness: unripe, medium ripe, and fully ripe [43]. The different ripeness classes correspond to different color characteristics and require a hardness [44] threshold within the corresponding range to enhance the discrimination confidence.

### 2.2. Fruit Ripening Test Platform

**Figure 2:**
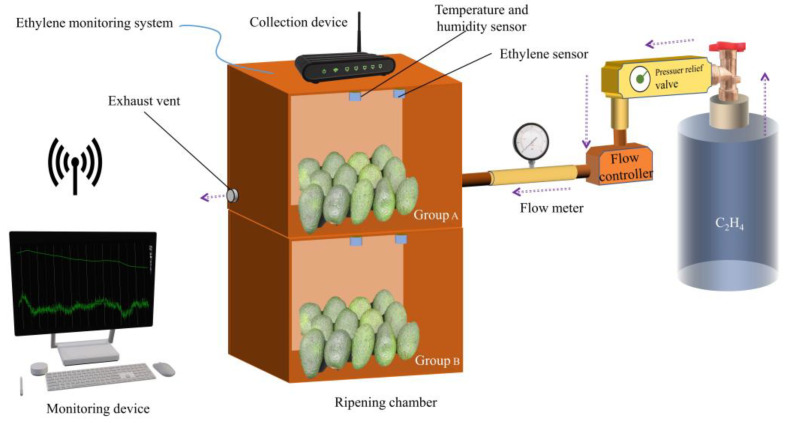
Test setup for ethylene sensor response experiment.

In this paper, the test platform device is a closed vessel with a size of 400 mm × 350 mm × 250 mm, with a gas inlet and outlet at both ends. The ethylene sensor was placed above the gas chamber, the ethylene monitoring system was placed outside the gas chamber, and the gas chamber inlet was connected to the standard ethylene gas high-pressure tank through the flow controller and pressure-reducing valve. The standard ethylene gas used was from Beijing Nanfei Gas Co., Beijing, China. The source of the sample was a Hass avocado from Mexico, with ripeness in the green fruit unripe state and inedible. The test setup is shown in Figure 2.

In this paper, two sets of tests were conducted with the ethylene monitoring system. Test 1 was a periodic test of the repetitive response of the ethylene sensor to a fixed concentration of standard ethylene gas, aiming to analyze the ethylene sensor’s repeatability and stability. The test was repeated for ten cycles for each test setup under the same test conditions. The second test was to verify the characteristics of the ethylene sensor for fruit ripening monitoring by releasing ethylene gas during the fruit ripening process.

**Figure 3:**
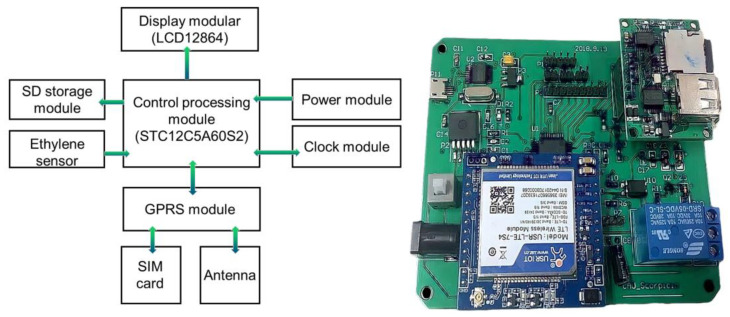
Ethylene sensor response experimental device diagram.

The control unit of this system adopts the STC12C5A60S2 microcontroller produced by Acerchip (a company based in Beijing, China) as the control chip; the output unit includes a storage module, wireless transmission module, and display module. The storage module uses a portable removable SD card storage; the wireless transmission module uses a 4G wireless transmission module (model: USR-LTE-7S4), and the display module uses an LCD12864 liquid crystal display. The input unit ethylene sensor was an electrochemical sensor with a three-electrode system produced by Anjeda (a company based in Beijing, China), model AJD-7C_2_H_4_. The AJD-7C_2_H_4_ is an intelligent ethylene module that uses electrochemical principles to detect the presence of ethylene gas in the air. A built-in temperature sensor allows for temperature compensation. It also has digital output and analog voltage output for ease of use. The module features high sensitivity, high resolution, low power consumption, high stability, and excellent anti-interference capability. The system hardware block diagram is shown in Figure 3.

## 3. Results and Discussion

### 3.1. Kinetic Analysis of Ethylene Sensor Response

**Figure 4:**
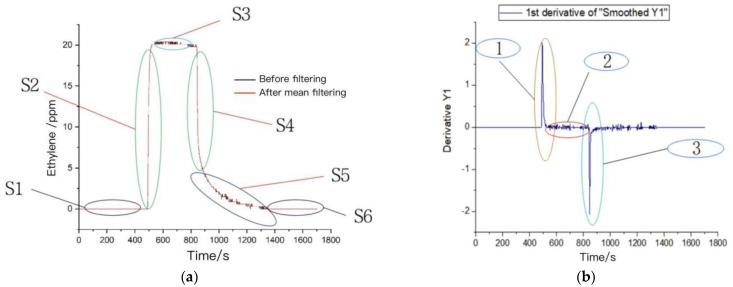

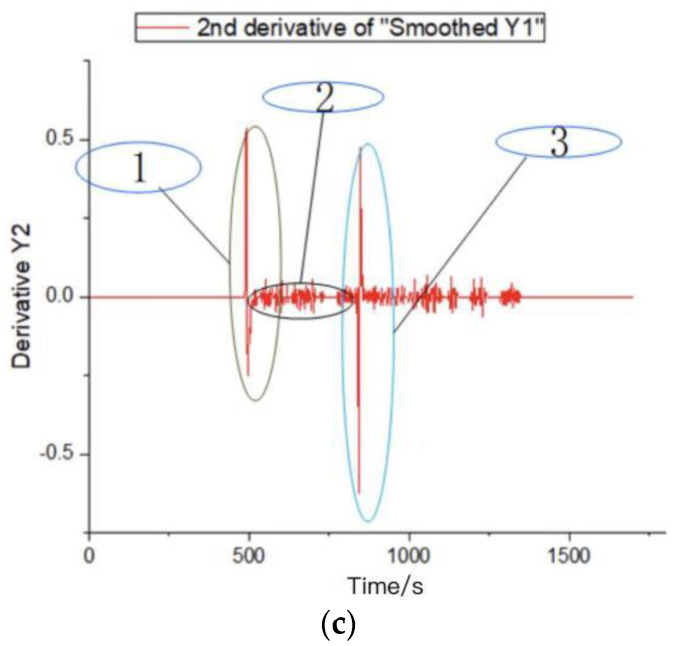
Typical response curve of ethylene sensor: (**a**) original response curve; (**b**) first-order derivative curve parameters; (**c**) second-order derivative curve parameters.

According to the electrochemical sensor principle, a chemical reaction occurs when the chemical in the sensor encounters the target gas ethylene, which leads to an increase in electric charge and voltage value. This linear relationship can characterize the change in ethylene gas concentration. A typical concentration curve of the ethylene sensor response and its first- and second-order derivative curves are given in Figure 4. From the time domain curve, it is clear that the ethylene gas sensor response is divided into a total of six stages, S1–S6.

The S1 stage is the response stage of the ethylene sensor in the air, and it can be seen from the curve that the response value of the ethylene sensor in the air is 0 ppm, which matches the actual atmospheric ethylene composition.

The S2 stage is the rapid rise stage, which passes 21 ppm of standard ethylene gas into the gas chamber of the test device at the rate of 500 mL/min through the flow control meter, from the time domain curve of S2 it can be seen that the ethylene gas sensor responds rapidly to ethylene gas, and the time taken to reach 70% of the maximum response value is 25 s.

The S3 is the dynamic stable response stage when the ethylene concentration in the gas chamber has reached the saturation state. The gas sensor value reaches a maximum of 20.4 ppm as seen in the S3 stage of the time domain curve, and is dynamically stable at 20.15 ppm, which shows that the sensor has good dynamic response stability.

The S4 stage is the rapid decline stage. At this stage, the test device gas chamber cover was opened to fully expose the sensor to the air, and the sensor value declines rapidly as seen in the S4 stage of the time domain curve.

The S5 phase is the slow recovery phase, wherein the sensor value decreases slowly. The possible reason for this may be that there remained a small amount of ethylene gas around the sensor equipment, impacting the sensor.

The S6 phase is the final recovery phase. The sensor value returns to the base concentration of 0 ppm in the air during this phase.

The response amplitude cumulative change, response change rate, response recovery rate, and acceleration cannot be obtained from the time domain characteristic curve, so the first- and second-order differentiations of the time domain curve were carried out to obtain the first- and second-order derivative plots in Figure 4b,c. From the first-order derivative curve, we can see that the maximum change speed in sensor response was 2.01714, and the minimum change speed was −2.07286. From the second-order derivative curve, the maximum acceleration of the sensor response was 0.53571, and the minimum acceleration of the sensor response was −0.62357.

### 3.2. Ethylene Sensor Feature Parameter Extraction

The test has examined repeatability and stability, the two aspects of analysis used for the extracted feature parameters and for determining from the feature parameters the subsets that can most accurately characterize the ethylene sensor’s properties. The feature parameters, selected from the test data measured under the same test conditions, should have a low noise level and less dispersion.

The responses of the characteristic parameters to different concentrations of the same gas should have linear proportional relationships with the concentration. According to these principles, the stability of typical parameters is characterized by a variation coefficient, and the proportion characterized by a correlation coefficient and a linear relationship.

**Figure 5:**
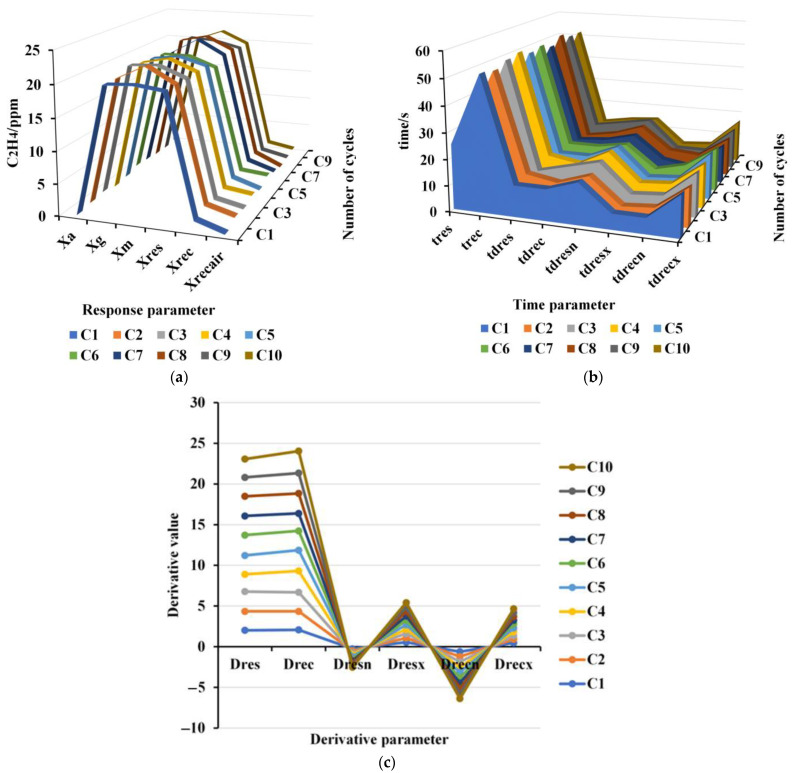
Response characteristics of ethylene sensor to 21 ppm C_2_H_4_: (**a**) repeatability of response parameters; (**b**) repeatability of time parameters; and (**c**) repeatability of derivative parameters.

The response characteristics of the ethylene sensor tested cyclically at room temperature are given in Figure 5. All data of the tests were measured under the same conditions, and the extracted characteristic parameters can be considered a normal distribution. Therefore, the mean value and the variance were calculated according to the coefficient variation formula, and all the corresponding coefficient variations were calculated. Statistically, a small variation indicates good stability of the characteristic parameters. Table 2 shows the statistical values of the ethylene concentration response characteristics. Xa, Xrec, and Xrecair are the typical parameters of the sensor in the air, and Xg, Xm, and Xres are the response values of the sensor in ethylene. The larger the difference between the value of the response characteristic in air and the value of the response characteristic in ethylene gas, the easier it is to be detected. The small coefficient of variation indicates that the characteristic parameters have good stability. Therefore, Xa was selected as the baseline value in air and Xg as the response characteristic in the measured gas.

The repeatability of the time response characteristics of the ethylene sensor is shown in Figure 5b. Table 3 presents the statistical analysis of the time response characteristics of the ethylene sensor and the coefficient of variation of the derivative information. Table 4 shows the statistical analysis of the correlation of the response-time characteristics parameters. The time information is an important parameter to describe the response characteristics of the sensor. Because of baseline drift, the time characteristics are more variable and less correlated with other quantities. Combined with Figure 5b and Table 3 and Table 4, the time response parameter tres and the recovery time parameter trec correlate more with other information. Therefore, tres and trec were selected as the time characteristic parameters. Table 5 gives the correlation statistics information on the derivative characteristics of the sensor response. It can be seen that the correlation coefficient between them is relatively low. Combined with Figure 5c and the coefficient of variation, the first-order derivative is better than the second-order derivative. Combined with the time parameter information, Dres and Drec were selected as the derivative feature information parameters. Table 6 gives statistical information on the integral characteristic parameters. It can be seen that the correlation between the integral characteristic and Xg was weak, therefore intT was selected as the information of the integral characteristic parameters. In summary, Xa, Xg, tres, trec, Dres, Drec, and intT were finally selected to form the feature subsets for characterizing the ethylene sensor.

### 3.3. Selection of Optimal Feature Parameters 

The ripening process in this experiment was as follows: ethylene gas was added to one group at the concentration of 21 ppm, which was marked as group A. To another group was added no ethylene gas but only air, which was marked as group B. Ten avocados were placed in each group. After one day, the ethylene gas was exhausted from the air-tight box A, and the ripening process was continued at the temperature of 20 °C. After one day, three avocados were randomly selected from each group and their ripening parameters were tested to obtain the variations.

**Figure 6:**
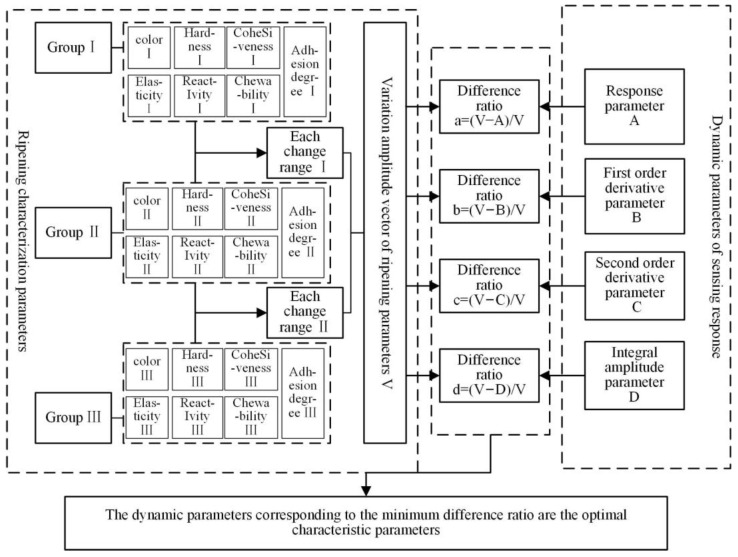
Optimal feature selection process for the sensing response dynamic.

The selection process of the optimal characteristic parameters for the whole experiment is shown in Figure 6, where the ripening characterization parameters and the sensing response kinetic parameters were obtained from the ripening test. The ripening parameter characteristics of the avocados on different days were processed to establish the change amplitude vector of the ripening parameters. Additionally, the change amplitude vectors of different parameters were obtained by analyzing the kinetic parameters of the sensing response. Then, the difference ratio between the variation amplitude vector of the ripening parameters and the variation amplitude vector of the different kinetic parameters was calculated to select kinetic parameters with the same variation trend as the optimal characteristic parameters.

**Figure 7:**
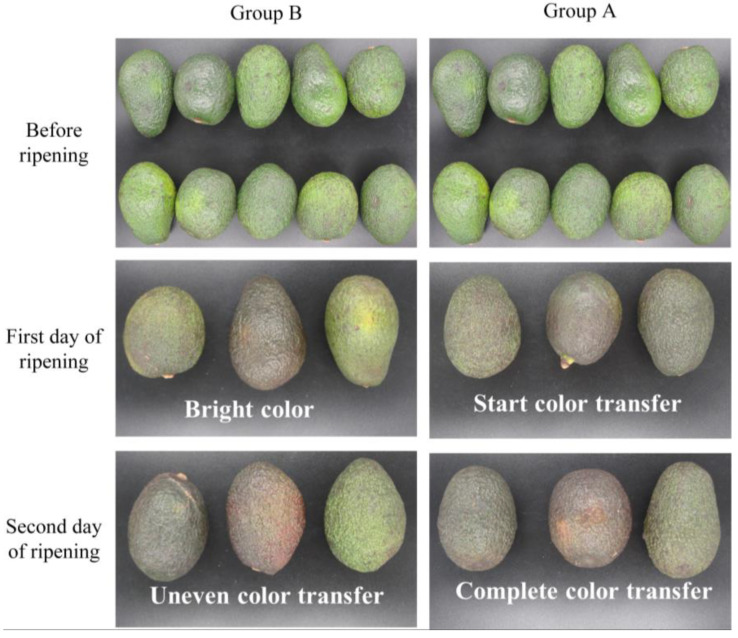
Color status of avocados during ripening.

**Figure 8:**
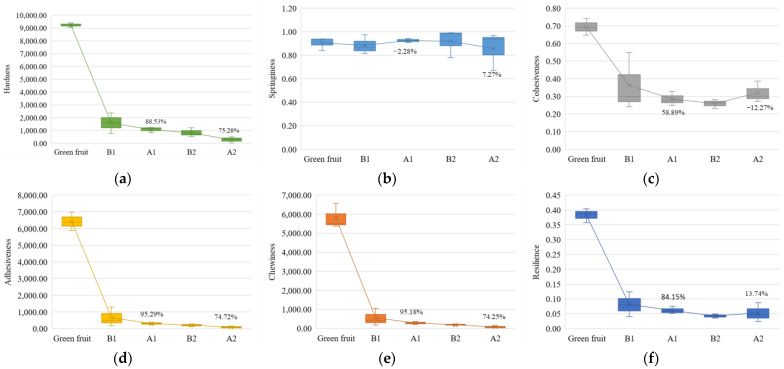
The box plot of texture analyzer metrics (B1 represents group B on the first day of ripening; A1 represents group A on the first day of ripening; B2 represents group B on the second day of ripening; and A2 represents group A on the second day of ripening): (**a**) hardness; (**b**) springiness; (**c**) cohesiveness; (**d**) adhesiveness; (**e**) chewiness; and (**f**) resilience.

First, the ripening characterization parameters during ripening were analyzed. The color characteristics of avocados under different ripening days are shown in Figure 7. The concrete hardness characteristics of avocados are shown in Table 7. Some other indicators of the texture analyzer are shown in Figure 8.

From the experimental data in Figure 7, it can be seen that, under the same temperature, the avocados of both groups A and B were bright green before ripening. After one day, the avocados of group A have no bright green color, while group B still has obvious brightness. On the second day, group A completely transformed its color, while group B still has obvious green color. Thus, it can be concluded that the avocados with the addition of ethylene gas made more color transformation than those without the addition of ethylene. From the test data in Table 7, it can be seen that the average hardness of avocados in group A was significantly lower than that in group B at the same time, and it can be concluded that the hardness of avocados with the addition of ethylene gas decreased faster. Thus, it can be concluded that ethylene could accelerate the ripening process of avocados.

The analysis focused on the changes in ripening characterization parameters in group A to investigate further the correlation between the characteristic parameters of ethylene and the ripening characterization parameters. By analyzing the color changes of the group A avocados in Figure 7, it can be seen that the area of color change on the first day of ripening accounts for almost half of the total area. On the second day of ripening, it turned color completely. By analyzing the change in hardness of the avocados in group A, as shown in Table 7, it can be concluded that the hardness on the first day of ripening was about 1/10 of the original hardness and approximately 1/30 on the second day of ripening. Figure 8 shows other indicators of the texture analyzer, including hardness, elasticity, cohesiveness, adhesion, chewability, and reactivity. Figure 8a shows hardness changed by 88.23% firstly and 75.28% secondly; Figure 8d shows adhesiveness changed by 95.29% firstly and 74.72% secondly; and Figure 8e shows chewiness changed by 95.18% firstly and 74.25% secondly. It can be concluded that the hardness, adhesiveness, and chewiness changed significantly during the ripening process, and that the rational index values of group A were smaller than those of group B. In summary, color, hardness, adhesiveness, and chewiness can be used as this experiment’s critical parameters for ripening.

**Figure 9:**
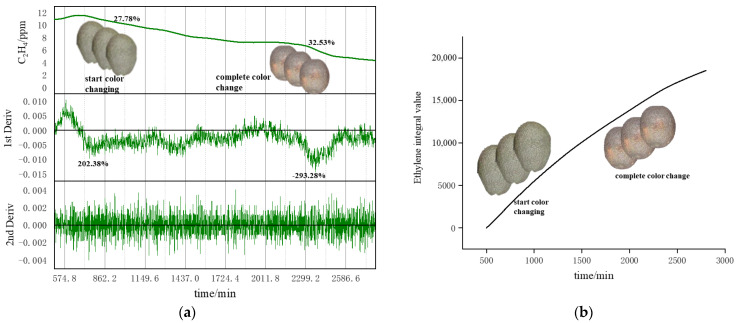
Ethylene parameter curve during ripening: (**a**) ethylene and its first and second-order derivative curves; (**b**) ethylene integration curve.

Figure 9 shows the changes in ethylene parameters monitored during the one-day ripening process. From Figure 9a, ethylene and its first- and second-order derivative curves are shown, from top to bottom; ethylene concentration curve, ethylene first-order derivative curve, and ethylene second-order derivative curve. The ethylene concentration curves show that the fruits started to release a large amount of ethylene after being excited by the exogenous application of ethylene and started to decline after rising to their peak. Then there were two periods of obvious decline with the change of time, indicating that the fruits’ physiological characteristics changed greatly during the ripening process. The ethylene concentration curve was further differentiated to find its first- and second-order derivative curves, in which the first-order derivative curve showed two changes. In contrast, the second-order derivative showed no obvious changes. By analyzing the first-order derivative curves, it was found that the first drop from peak occurred in the 600–700 min period, and the second occurred in the 2200–2400 min period. This indicates that these were the critical points and had a strong correlation with the various characteristics of the ripening characterization parameters. Figure 9b shows the ethylene integral curve, from which it can be analyzed that the ethylene release rate from the fruit gradually slows down with time, indicating that the fruit’s intrinsic physiological characteristics change and affect the release of endogenous ethylene, which has a certain correlation with the ripening characterization parameters of the fruit. According to the optimal parameter selection process shown in Figure 6, the final optimal characteristic parameters are the response and first-order derivative parameters.

## 4. Conclusions

The paper aimed to analyze the response characteristics of a customized ethylene sensor for fruit ripening and to explore the feasibility of its practical application in the face of the needs of the ripening process (high precision, small range).

The results show that, firstly, the monitoring system has good stability and is suitable for ethylene monitoring. Secondly, the characteristic parameters of the ethylene gas sensor and ripening process could be described by typical parameters. The coefficient variation of typical parameters, such as air response base value Xa, steady-state response value Xg, response time tres, response recovery time trec, response speed Dres and response recovery speed Drec, were all below 4.69%, and the response amplitude integration intT had the lowest correlation with Xg. Finally, the ethylene gas sensor has good performance in response speed, stable response, sensitivity and stability, and can play an effective role in monitoring ethylene during the actual ripening process. The sensor can accurately identify ethylene gas, monitor its changes in the ripening process and reflect the ripening state of fruits.

## Figures and Tables

**Table 1:** Characteristic parameters and their description.

Type	Features	Description
Response parameters	Xa	Baseline values in air
	Xg	Steady-state response values in the measured gas
	Xm	Maximum response value in the measured gas
	Xres	Response value when placed in the measured gas for 1 min
	Xrec	Recovery value at 3 min after taking out the measured gas
	Xrecair	Final recovery value
First-order derivative	Dres	Maximum value of the first-order derivative of response (abs. value)
	Drec	Recover the maximum value of the first-order derivative (abs. value)
Second-order derivative	Dresn	Minimum second-order derivative response value
	Dresx	Maximum second-order derivative response value
	Drecx	Maximum second-order derivative recovery value
	Drecn	Minimum second-order derivative recovery value
Time parameters	tres	Response time
	trec	Recovery time
	tDres	Time from gas entry to Dres
	tDrec	Time from gas out to Drec
	tDresn	Time from gas entry to Dresn
	tDresx	Time from gas entry to Dresx
	tDrecn	Time from gas out to Drecx
	tDrecx	Time from gas out to Drecn
Integral parameters	intT	Integration from gas entry to exit time period
	IntPres	Response integral from gas entry to the moment t res
	IntPrec	Recovery integral from gas out to the moment t rec

**Table 2:** Statistical analysis of ethylene concentration response characteristics.

	Xa	Xg	Xm	Xres	Xrec	Xrecair
Mean value/ppm	0.388	20.6	21.5	19.81	1.87	0.74
Coefficient of variation/%	2.21	2.42	3.54	2.84	16.32	57.76

**Table 3:** Statistical analysis of response time and derivative information.

Type	Average Value	Coefficient of Variation/%
**tres**	26.7	4.69
**trec**	52.4	2.05
**tdres**	12.3	7.71
**tdrec**	12.9	9.28
**tdrean**	16.3	6.5
**tdresx**	6.2	14.82
**tdrecn**	6.7	17.31
**tdrecx**	16.3	6.50
**Dres**	2.31	3.28
**Drec**	2.41	3.51
**Dresn**	−0.25	−5.22
**Dresx**	0.54	6.31
**drecn**	−0.64	−3.29
**drecx**	0.47	4.52

**Table 4:** Correlation analysis of time information.

	tres	trec	tdres	tdrec	tdresn	tdresx	tdrecn	tdrecx
**tres**	1							
**trec**	−0.06606	1						
**tdres**	0.364932	−0.02179	1					
**tdrec**	0.42264	0.120869	−0.26414	1				
**tdresn**	0.075418	0.565914	0.011056	0.113891	1			
**tdresx**	0.251164	0.584898	−0.20393	0.020199	0.616348	1		
**tdrecn**	−0.14546	0.730978	0.292929	−0.10405	0.17187	0.166848	1	
**tdrecx**	−0.51116	0.2732	0.121616	0.113891	0.207921	−0.29676	0.53370	1

**Table 5:** Derivative information feature correlation.

	Dres	Drec	Dresn	Dresx	Drecn	Drecx
**Dres**	1					
**Drec**	0.071136	1				
**Dresn**	−0.49589	0.276845	1			
**Dresx**	0.202957	0.416269	0.06747	1		
**Drecn**	−0.65594	−0.01297	0.297729	−0.10284	1	
**Drecx**	−0.02493	0.38014	−0.38383	0.288566	−0.13551	1

**Table 6:** Integral information feature correlation.

	Xg	intT	intPres	intPrec
**Xg**	1			
**intT**	0.12053	1		
**intPres**	−0.6605	−0.29101	1	
**intPrec**	0.123797	−0.27378	0.114264	1

**Table 7:** Comparison of hardness during ripening (unit: grams).

Sample	Before Ripening	First Day of Ripening		Second Day of Ripening	
		B	A	B	A
Sample A	9225.428	772.574	800.262	702.202	492.847
Sample B	9070.682	2360.329	1145.055	509.614	286.187
Sample C	9406.169	1645.132	1232.887	759.652	6.768

## Data Availability

Not applicable.
